# Refining macrostomia correction: Case series applying square flap technique and Z/W-plasty skin closure for enhanced aesthetic and functional outcome

**DOI:** 10.1016/j.ijscr.2023.109023

**Published:** 2023-11-10

**Authors:** Ali Sundoro, Dany Hilmanto, Hardisiswo Soedjana, Ronny Lesmana, Rani Septrina, Lisa Y. Hasibuan, Graciella Novian Triana Wahjoe Pramono

**Affiliations:** a Division of Plastic Reconstructive and Aesthetic Surgery, Department of Surgery, Faculty of Medicine Universitas Padjadjaran - Dr. Hasan Sadikin General Hospital, Bandung, Indonesia; b Department of Child Health, Faculty of Medicine, Universitas Padjadjaran - Dr. Hasan Sadikin Hospital, Bandung, Indonesia; c Department of Basic Medical Science, Faculty of Medicine, Universitas Padjadjaran, Bandung, Indonesia

**Keywords:** Macrostomia, Facial cleft, Commisuroplasty, Z-plasty, W-plasty

## Abstract

**Introduction and importance:**

Macrostomia is a congenital deformity found in Tessier no. 7 facial clefts defined as an enlargement of the mouth at the oral commissure. Several techniques are described in literature to achieve optimal functional and aesthetic results, with varying results and surgeon preferences. In this case series we report surgical repair of macrostomia with a vermillion square flap method for the oral commissure combined with either Z-plasty or W-plasty closure for the skin.

**Cases presentation:**

A retrospective case analysis of 12 patients with macrostomia operated over the past 7 years at our plastic surgery division was performed (by two different operators; 11 cases by A.S. and 1 case by R.S.). Clinical features of the patients were analyzed through photography documentation, and patient description such as age of operation, operation technique, and complications were obtained through patient records. Macrostomia was corrected with a vermillion square flap method for commissure, overlapping muscle closure, along with either Z-plasty or W-plasty closure for the skin. Quality of lip commissure position, symmetry, thickness of vermillion, and scar result were recorded.

**Clinical discussion:**

In all twelve patients repaired with the overlapping muscle closure and square flap, the lip commissures were formed with satisfactory shape, position, and thickness with no commissure contracture during the follow up period. The Z-plasty was a simpler method compared to the W-plasty, and resulted in comparable scars. One patient (adult with hemifacial macrostomia and W-plasty skin closure) underwent revision surgery for more accurate symmetry and position of the oral commissure.

**Conclusion:**

There are many varieties of surgical repair for macrostomia, and each method should be adjusted and combined according to each patient. Overall, macrostomia repair with this technique combination produced satisfactory aesthetic and functional results in all twelve patients. Z-plasty for skin closure after muscle and vermillion closure was a simpler technique and resulted in comparable scars than W-pasty closure in this case series.

## Highlights

• Macrostomia is defined as an enlargement of mouth at the oral commisure
• Operative goals for macrostomia include functional repair dan enhanced aesthetic
• Square flap and Z/W-plasty skin closure will reduced contractile scar and minimize the visible scar

## 1 Introduction

Macrostomia is a rare congenital deformity. Also called the ‘transverse facial cleft’, it is defined as an enlargement of the mouth at the oral commissure [1,2]. This deformity may occur as an enlargement at one or both of the oral commissures, with an incidence rate between 1 in 60,000 to 1 in 300,000 live births [3,4].

Macrostomia results from a failure of fusion of the maxillary and the mandibular processes, and may occur isolated or with other congenital abnormalities. Associated abnormalities or syndromes include the Treacher-Collins syndrome, Goldenhar syndrome, hemifacial microsomia, bone anomalies, abnormalities of the middle and external ear, as well as preauricular skin tags [1,5].

To manage this deformity, surgeons have developed their own surgical and repair preferences [3,6]. In 1969 Boo-Chai proposed the use of landmarks for the positioning of the new oral commissure (two pillars of muscle bordering the sides and the change in the colour of the vermillion), and the suturing of the free ends of the orbicularis oris exactly at the commissure to prevent a ‘gold-fish mouth appearance’. [6,7] In 1981, a different technique was proposed by Kaplan to create a neocommissure using an upper lip vermilion flap that is transposed to the lower lip. This approach resulted in a commissure with no suture line or scar, avoiding contracture, deformity and pain with mouth movement [3,8]. For skin closure, the most commonly used methods include straight-linear closure, Z plasty, or W plasty [3,5,9].

Operative goals for macrostomia include functional repair of the orbicularis oris, commisuroplasty without contractile scar and skin, and buccal mucosal closure of the excised cleft with a minimally visible scar [6,10]. We describe our operative procedure and representative patients, with quality of lip commissure position, symmetry, vermilion thickness, and scar result as our output parameter.

## 2 Presentation of cases

A retrospective case analysis of 12 patients with macrostomia operated over the past 7 years at our plastic surgery division was performed (by two different operators; 11 cases by A.S. and 1 case by R. S, both are certified plastic surgeons). Patients were diagnosed with macrostomia and required repair of their deformity. Regarding syndromic associations, none were identified in any of the patients included in our study. Our investigation was centered on unilateral cleft lip without concurrent syndromic conditions (Fig. 4, Fig. 5, Fig. 6, Fig. 7, Fig. 8, Fig. 9, Fig. 10, Fig. 11, Fig. 12, Fig. 13).

Clinical features of the patients were analyzed through photography documentation, and basic demographic information (age during operation, sex, operation technique, and complications) were obtained through patient records. The output parameter after macrostomia repair was quality of lip commissure position, symmetry, thickness of vermillion, and scar result.

The informed consent process was conducted in accordance with established ethical guidelines and institutional policies. It involved a detailed explanation of the surgical procedure, its potential risks and benefits, and the importance of photographic documentation for medical records. Additionally, the possibility of publishing anonymized clinical data for scientific and educational purposes was thoroughly discussed. Subsequently, written consent was obtained from each party, signifying their understanding and approval of the outlined procedures.

The work has been reported in line with the PROCESS criteria [11].

### 2.1 Surgical technique

**Fig. 1:**
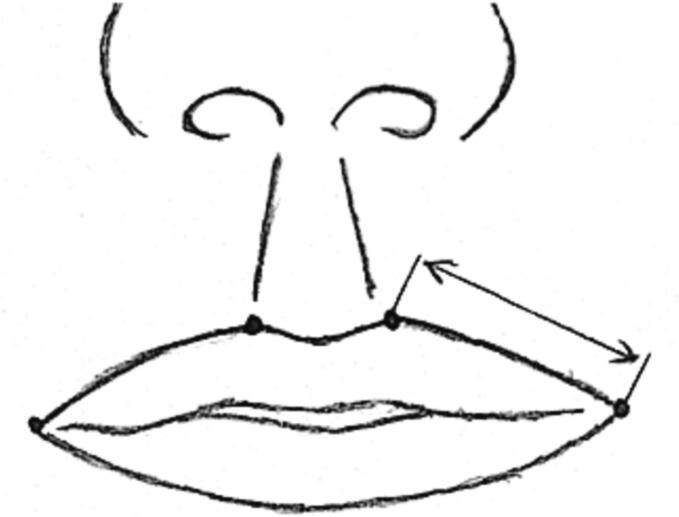
Distance of the normal commissure to the philtrum was measured and consequently marked on the contralateral vermillion-cutaneous junction.

**Fig. 2:**
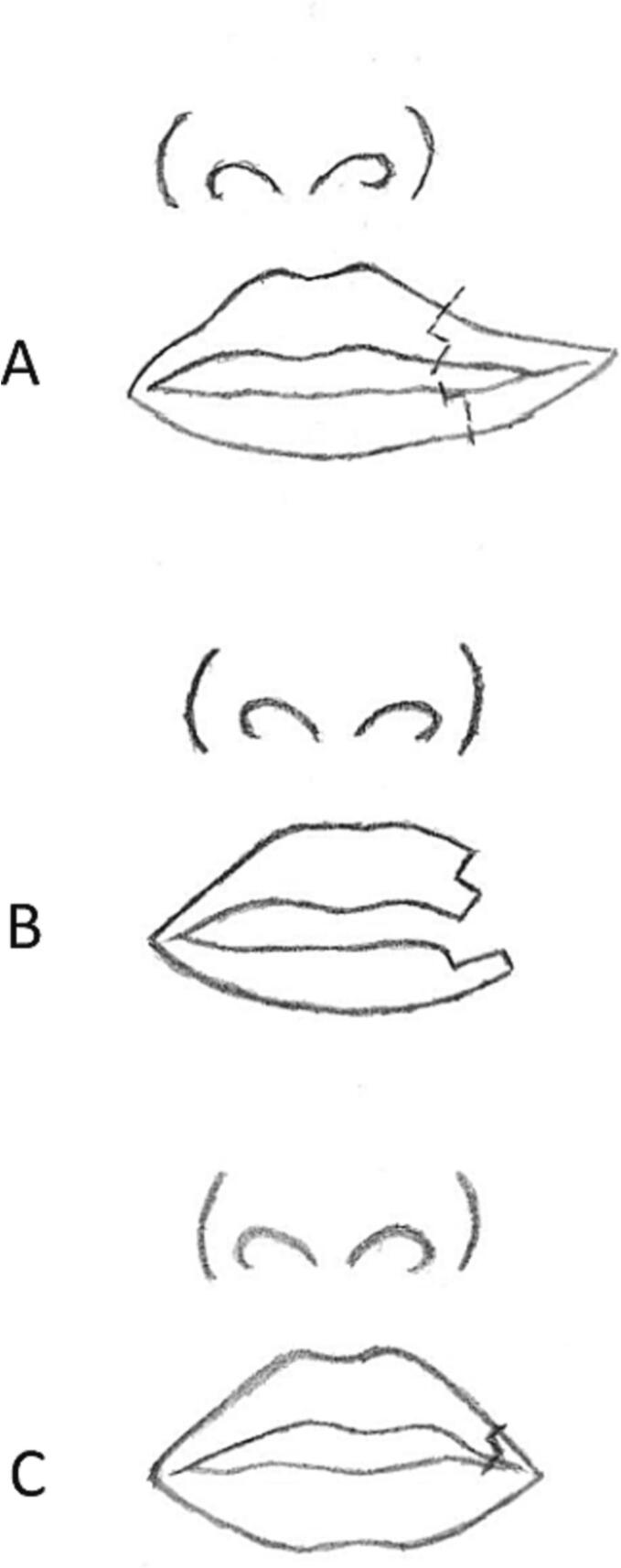
(a) preoperative markings on the upper and lower vermillion (b) design of vermillion square flap of upper lip (c) final closure.

Reconstruction was done under general anaesthesia and with endotracheal intubation positioned on the midline in all the patients. The determination of anatomic position for the new oral commissure depended on whether the patient had unilateral or bilateral macrostomia. The new oral commissure position for unilateral macrostomia was determined and adjusted according to the normal contralateral commissure. Distance of the normal commissure to the philtrum was measured and consequently marked on the contralateral vermillion-cutaneous junction (Fig. 1), then anatomic position was re-confirmed by dropping a vertical line from midgaze. Patients with bilateral macrostomia had their new commisures determined by dropping a vertical line from medial limbus at midline gaze and marking the vermillion-cutaneous junction of the upper and lower lip. Markings form a vermillion square flap on the mucocutaneous border of the lower lip that will eventually be sutured to the top lip's mucocutaneous border (Fig. 2).

After injecting a small amount of 0.5 % lidocaine with 1: 200,000 epinephrine solution, incisions are made, and the skin and mucosa are dissected to expose the orbicularis oris muscle. The excess tissue of the buccal mucosa is excised and sutured in a linear fashion using an absorbable suture (monofilament polyglycolic acid PGA 5–0). The orbicularis oris upper and lower muscle bundles are identified, then sutured in an overlapping fashion using absorbable suture (monofilament PGA 5/0) with the top bundle placed anterior to the lower bundle using a horizontal matress suture.

**Fig. 3:**
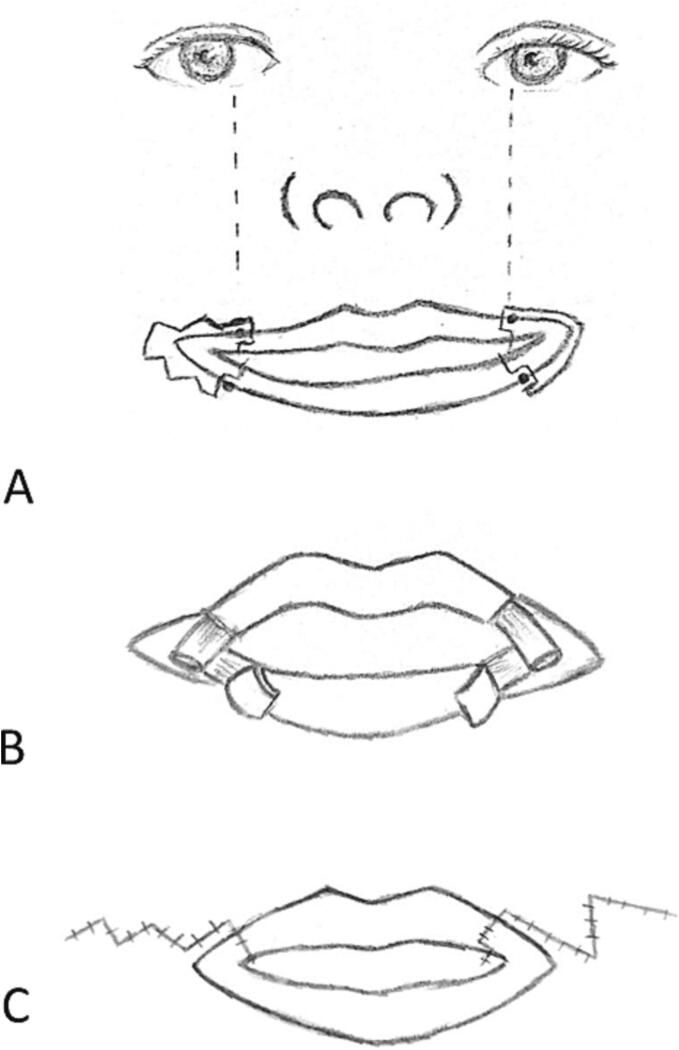
(a) Diagram of preoperative planning on a bilateral macrostomia, with an example of W-plasty planned on the right side (b) Muscle bundles are identified and sutured in an overlapping fashion using horizontal mattress suture (c) The skin appearance when closed with a W-plasty on the right side and a Z-plasty on the left side.

Regarding skin closure, the twelve patients were then divided into two groups using different methods: Z-plasty and W-plasty. Six patients with skin closure using W-plasty had the design incised at the start of the procedure along with the flap (Fig. 3). As for the six patients with skin closure using Z-plasty, the design was marked after suture of the mucosa and muscle. The Z-plasty is designed according to the length of the skin incision. For both W- and Z-plasty, simple interrupted 6/0 nylon sutures were utilized.

**Fig. 4:**
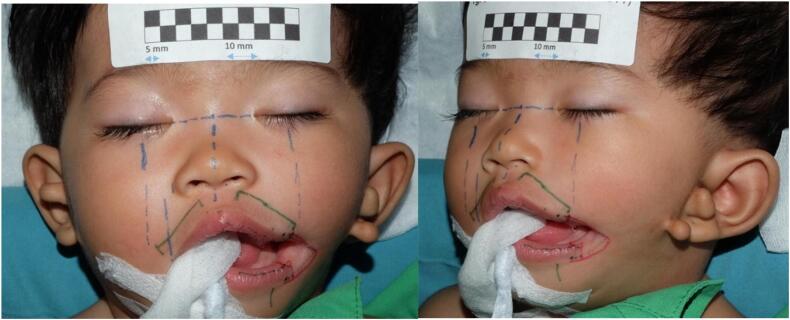
Preoperative planning on a patient with unilateral macrostomia.

**Fig. 5:**
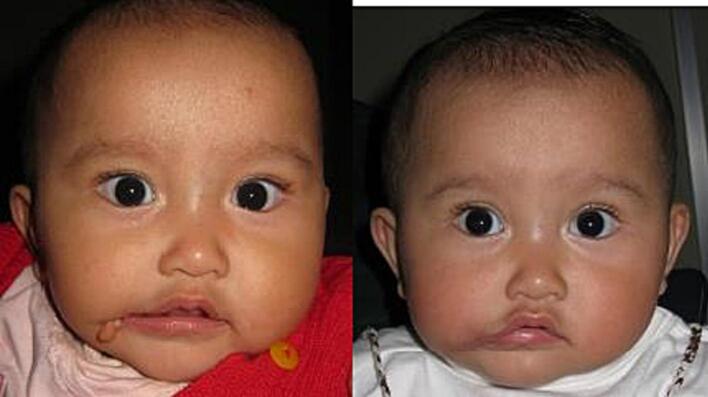
Patient 1 – Preoperative and 2 weeks postoperative image.

**Fig. 6:**
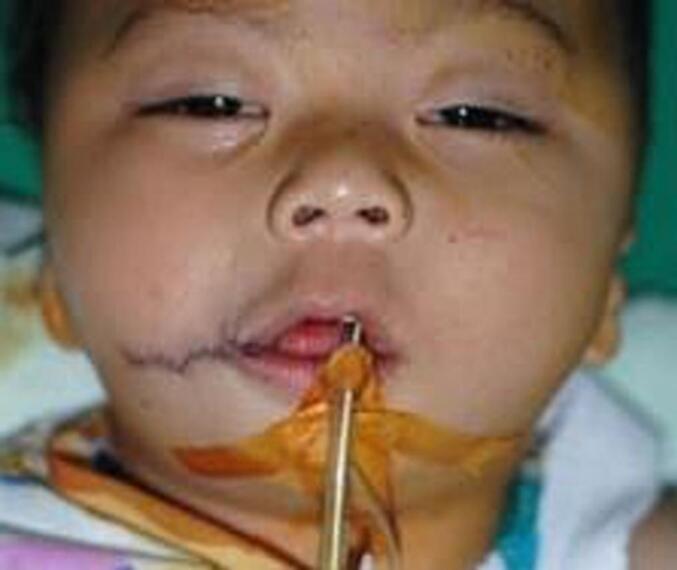
Patient 2 – Directly postoperative with W-plasty skin closure.

**Fig. 7:**
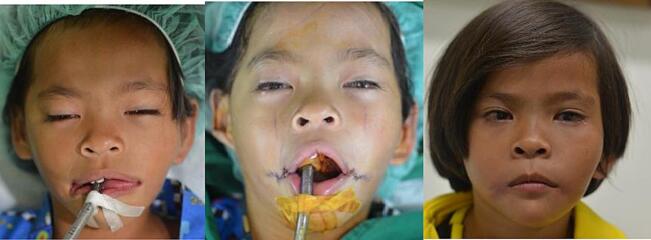
Patient 3 – Preoperative, directly postoperative and 1-month postoperative image.

**Fig. 8:**
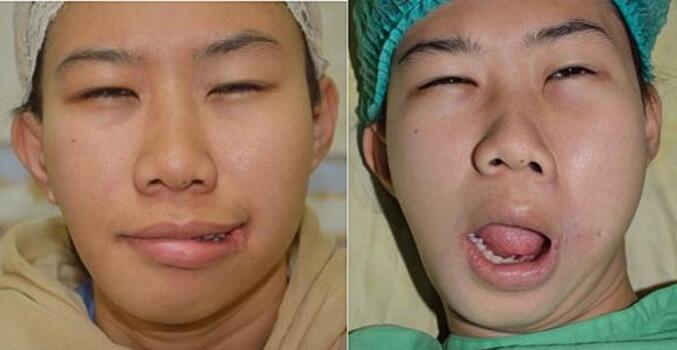
Patient 4 – Preoperative and 6 months postoperative image. This patient then underwent a revision surgery for re-positioning of the left commissure.

**Fig. 9:**
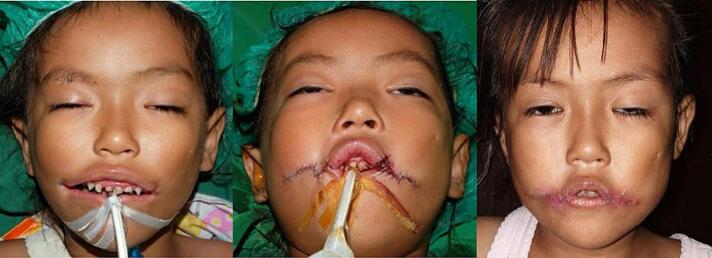
Patient 8 – Preoperative, directly postoperative, and 1 month postoperative image.

**Fig. 10:**
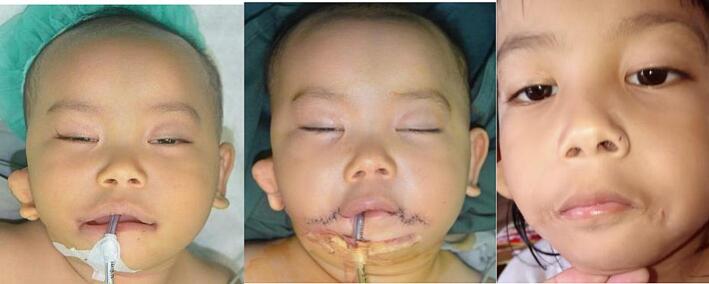
Patient 9 – Preoperative, directly postoperative, and 3 years postoperative image.

**Fig. 11:**
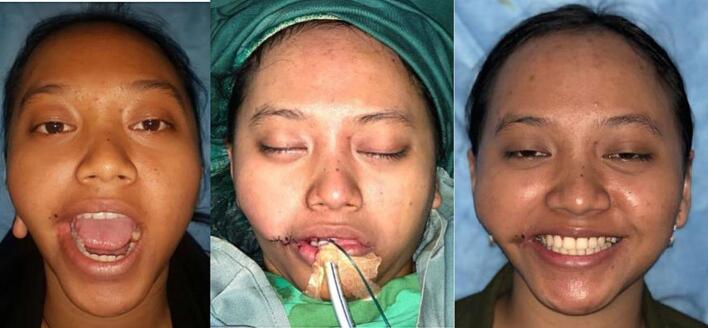
Patient 10 – Preoperative, directly postoperative, and 1 week postoperative image.

**Fig. 12:**
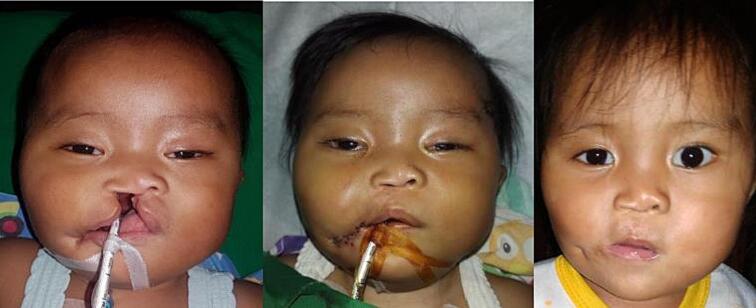
Patient 11 – Preoperative, directly postoperative, and 2 years postoperative image.

**Fig. 13:**
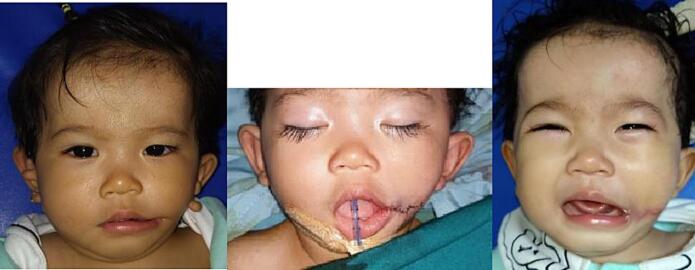
Patient 12 – Preoperative, directly postoperative, and 1 week postoperative image.

After surgery, the wound was covered using impregnated gauze, gauze, and adhesive tape. The patient was educated to maintain the wound dressing for 24 h, then they should clean and treat the wound by applying 1 % Chloramphenicol eye ointment around five to six times per day. The patient was then discharged and told to visit the outpatient clinic for skin suture removal 7 days after the operation.

## 3 Results

Twelve patients underwent surgery; eight female and four male patients. All patients were operated using the vermillion square flap technique, but for the skin closure half patients underwent W-plasty and the other half Z-plasty. There was a wide age range; the youngest patient that underwent surgery was 4 months old, and the oldest 25 years old. Table 1 presents clinical data of all the patients.

**Table 1:** Clinical characteristics of the patients.

No.	Gender	Age at surgery	Cleft	Associated anomalies	Skin closure	*Re*-operation
1	Female	10 months	Unilateral (R)	Accessory tragus, skin tag	W-plasty	
2	Male	10 months	Unilateral (R)	–	W-plasty	
3	Female	9-year-old	Bilateral	Accessory tragus	W-plasty	
4	Female	25-year-old	Unilateral (L)	Accessory tragus, hemifacial microsomia	W-plasty	Yes
5	Male	11 months	Unilateral (L)	–	W-plasty	
6	Female	1-year-old	Bilateral	–	Z-plasty	
7	Male	4 months	Unilateral (L)	Ipsilateral accessory tragus	Z-plasty	
8	Female	4-year-old	Bilateral	–	Z-plasty	
9	Male	11 months	Bilateral	Unilateral right microtia	Z-plasty	
10	Female	17-year-old	Unilateral (R)	Accessory tragus, skin tag	Z-plasty	
11	Female	11 months	Unilateral (R)	Cleft lip	W-plasty	
12	Female	11 months	Unilateral (L)	Accessory tragus, hemifacial microsomia	Z-plasty	

The lip commissure of all twelve patients repaired with the overlapping muscle closure and square flap were formed with satisfactory shape, position and thickness with no commissure contracture. One patient (adult with hemifacial macrostomia and W-plasty skin closure) underwent revision surgery for more accurate symmetry and position of the oral commissure. One patient (child with bilateral macrostomia and Z-plasty closure) seemed to require a revision surgery for re-positioning of the oral commissures because the placement was too medial and mouth opening function should be reanalyzed. However, this patient was uncontactable for follow up. Overall, the Z-plasty was a simpler method, but scar results from W-plasty and Z-plasty yielded similar end results. None of the patients suffered from commissure contracture during the follow up period. There was no complaint regarding feeding. Mouth opening was satisfactory and lip seal was adequate for all patients.

## 4 Discussion

Macrostomia is a rare congenital deformity, the ‘transverse facial cleft’ defined as an enlargement of the mouth at the oral commissure [1,2]. This deformity may occur as an enlargement at one or both of the oral commissures of the mouth, with an incidence rate between 1 in 60,000 to 1 in 300,000 live births [3,4]. Associated abnormalities or syndromes include the Treacher-Collins syndrome, Goldenhar syndrome, hemifacial microsomia, bone anomalies, abnormalities of the middle and external ear, as well as preauricular skin tags [1,5].

Operative goals for macrostomia include functional repair of the orbicularis oris, commisuroplasty without contractile scar and skin, and buccal mucosal closure of the excised cleft with a minimally visible scar [6,10]. Various types of surgical repair have been applied to macrostomia repair, and one of the first techniques used in 1962 was an Estlander flap that used a full-thickness vermillion flap from the lower lip [5,7]. Further macrostomia repair development then led to the use of layered closure techniques for better placement of the muscle and oral commissure [7].

In 1969, Boo-Chai points out details for commissure placement, locating important landmarks (line of demarcation in the vermillion) and to place the muscle together as close to the new commissure as possible to avoid a ‘goldfish mouth’ appearance [7]. Kaplan then concludes and combines the methods used to determine commissure position by measuring the length of the upper lip on the normal side, noting the anatomical landmarks of the vermillion and medial limbus gaze, as well as using a standard lip measurement table [8,12]. This method was used in our study, where the new commissure was carefully formed based on the distance between the normal commissure and the philtrum. Patients with bilateral macrostomia had their new commissures based on dropping a vertical line from medial limbus on midline gaze.

In 1981, the vermillion commissure flap for commisuroplasty and overlapping muscle bundles for myoplasty was introduced by Kaplan due to Onizuka's observation that the commissure is not a corner but a rounding, continuous vermillion web [12,13]. The vermillion commissure flap is formed with its base at the lower lip to avoid a corner scar. Eguchi et al. then modifies the Kaplan technique, by placing the flap on the upper lip, rather than the lower lip. He hypothesized that a scar on the lower lip may become more conspicuous over time because of the tension created when the mouth is opened [14]. In this study, we applied the modified method proposed by Eguchi, where a vermillion square flap was created to form the commissure, but placed on the upper vermillion instead of the lower. Myoplasty applied in this method, where the upper stump was sutured anterior to the lower stump formed normal vermillion contour and thickness in all twelve patients.

The most common method for skin closure used in commisuroplasty is by Z-plasty of W-plasty, to create an inconspicuous scar and to avoid displacement or contracture. This is described by Kaplan, who also closes the skin with a z-plasty instead of linear closure to avoid lateral displacement [12]. Straight line closure is believed to result in contracture and lateral displacement, although actual studies have not proven this [3,6,15]. Some reports have presented results with inconspicuous scars after straight line closure [8,15]. In this study, half of the patients underwent skin closure with Z-plasty and the other half with W-plasty. The two techniques showed comparable scar results, with Z-plasty being the simpler method to perform. Yu et al. described that a more favorable scar would be achieved if the medial limb of the Z-plasty was made perpendicular to relaxed skin tension lines [9].

Limitations in this study include a short and inconsistent follow-up period. Patients are often uncontactable, or has moved to a different city due to the fact that our center is the main one in West Java. Further follow up should be planned, with a multidisciplinary team to monitor function and facial growth. Follow up surgery should be prepared if required.

## 5 Conclusion

There are many macrostomia repair techniques described in literature, with various results. We conclude that the myoplasty and vermillion square flap method creates a normal looking commissure and vermillion. Skin closure with W-plasty and Z-plasty yields similar scar results, with Z-plasty being the simpler method to perform.

## Funding

There was no any financial disclosure or support for this study.

### CRediT authorship contribution statement

Ali Sundoro: Study concept or design, data collection, data analysis, interpretation, writing the paper.

Dany Hilmanto: Study concept or design, data collection, data analysis, interpretation, writing the paper.

Hardisiswo Soedjana: Study concept or design, data collection, data analysis, interpretation, writing the paper.

Ronny Lesmana: Study concept or design, data collection, data analysis, interpretation, writing the paper.

Rani Septrina: Study concept or design, data collection, data analysis, interpretation.

Lisa Y. Hasibuan: Study concept or design, data collection, data analysis, interpretation.

Graciella Novian Triana Wahjoe Pramono: Study concept or design, data collection, data analysis, interpretation, writing the paper.

## Ethical approval

Ethical approval for this study (Ethical Committee N° NAC 207) was provided by

the Ethical Committee of Dr. Hasan Sadikin General Hospital, Bandung,

Indonesia on 20 October 2023

## Registration of research studies

This study does not require registration.

## Consent

Written informed consent was obtained from the patient's parents/legal guardian for

publication and any accompanying images. A copy of the written consent is available

for review by the Editor-in-Chief of this journal on request

## Guarantor

Ali Sundoro

Dany Hilmanto

Hardisiswo Soedjana

Ronny Lesmana

Rani Septrina

Lisa Y. Hasibuan

Graciella Novian Triana Wahjoe Pramono

## Declaration of competing interest

The authors declare no conflicts of interest related to this study.
